# BMAL1 alleviates sepsis-induced acute kidney injury by inhibiting apoptosis, ferroptosis and inflammation

**DOI:** 10.1186/s41065-025-00583-5

**Published:** 2025-10-14

**Authors:** Zhipan Chen, Gaoze Chen, Jinhui Shi, Litong Jin

**Affiliations:** Emergency Department, Taizhou Central Hospital (Taizhou University Hospital), No. 999, Donghai Avenue, Economic Development Zone, Taizhou, 318000 China

**Keywords:** USP10, HOXA5, Acute kidney injury, Ferroptosis, Sepsis, BMAL1

## Abstract

**Background:**

Sepsis is a life-threatening syndrome characterized by organ dysfunction. The kidney is one of the earliest organs to be injured during sepsis. Basic Helix-Loop-Helix ARNT Like 1 (BMAL1) was shown to play a critical role in immune responses. BMAL1 deregulation is related to sepsis-induced injury. Thus, correct understanding of the molecular mechanism of BMAL1 in sepsis-induced acute kidney injury (AKI) may be importance for seeking effective targeted therapy.

**Methods:**

Lipopolysaccharide **(**LPS)-induced renal tubular epithelial cells (HK-2 cells) and a sepsis-AKI model established in C57BL/6 mice using cecal ligation and puncture (CLP) were used for functional analyses. In vitro analyses were conducted using EdU assay, flow cytometry, MTT assay and ELISA, respectively. Levels of mRNA and protein expression were using qRT-PCR and western blotting. Cellular ubiquitination analyzed the ubiquitination effect of USP10 on BMAL1. The binding of HOXA5 to BMAL1 promoter was verified using Chromatin immunoprecipitation and Luciferase reporter assays.

**Results:**

BMAL1 overexpression reversed LPS-induced apoptosis, inflammation and ferroptosis in HK-2 cells, as well as attenuated sepsis-induced AKI in mouse models. Mechanistically, USP10 bound to BMAL1 and positively modulated BMAL1 expression by reducing BMAL1 ubiquitination. In addition, HOXA5 induced BMAL1 transcription. Moreover, USP10 or HOXA5 overexpression reversed LPS-induced apoptosis, inflammation and ferroptosis in HK-2 cells, which could be rescued by BMAL1 decrease.

**Conclusion:**

BMAL1 overexpression mediated by USP10-induced deubiquitination or HOXA5-induced transcription can attenuate sepsis-induced acute kidney injury, recommending a novel insight for the prevention of sepsis-induced AKI.

**Supplementary Information:**

The online version contains supplementary material available at 10.1186/s41065-025-00583-5.

## Introduction

Sepsis is a life-threatening syndrome that occurs when the body’s immune system has an extreme response to infection, characterized by organ dysfunction [1]. It is a major cause of deaths, there were 11 million sepsis-related deaths worldwide in 2020, accounting for 20% of the total global deaths [2]. The kidney is one of the earliest organs to be injured during sepsis that markedly enhances the mortality rates [3, 4]. Therefore, further better understanding the mechanisms by which acute kidney injury (AKI) during sepsis will benefit for developing and improving the therapies for sepsis-AKI.

Basic Helix-Loop-Helix ARNT Like 1 (BMAL1, also call ARNTL) is a circadian clock gene that modulates RNA transcriptional activation [5]. It has been reported to be implicated in diverse disorders, such as skeleton disorders, periodontitis, COVID-19 and cancers [6–8]. As a major circadian clock regulator, BMAL1 plays a critical role in both adaptive and innate immune responses, and BMAL1 deregulation is related to immune-associated diseases, including sepsis [9, 10]. Moreover, Tang et al. showed that increase of BMAL1 could rescue myocardial damage in sepsis via enhancing mitophagy through SIRT1 [11]. BMAL1 down-regulation in macrophages increased the CXCL2 expression to exacerbate sepsis-induced lung injury via promoting innate immunity and systemic inflammation [12]. In addition, BMAL1 overexpression alleviated renal tubular injury by suppressing ferroptosis through the reduction of YAP expression and the inactivation of the Hippo pathway [13], suggesting the potential involvement of BMAL1 in sepsis-induced AKI. However, the upstream molecular mechanism of BMAL1 in regulating sepsis-induced AKI remain unclear.

Herein, this study used lipopolysaccharide (LPS)-induced cell models in vitro and established septic mouse models in vivo to investigate the functions of BMAL1 in kidney injury during sepsis, moreover, the associated upstream molecular mechanisms were also explored, which may offer a novel insight into the prevention of sepsis-AKI.

## Materials and methods

### Cell culture and treatment

HK-2 cells were obtained from Procell (Wuhan, China) and cultured in DMFM/F-12 (Procell) plus 10% FBS and 1% penicillin-streptomycin (Beyotime, Beijing, China) with 5% CO2 at 37℃. For in vitro model, HK-2 cells were treated with 10 µg/mL LPS for 24 h.

### Vector construction

The full-length of BMAL1, ubiquitin-specific peptidase (USP) 10 or HomeoBox A5 (HOXA5) was cloned into pcDNA3.1 plasmids (GenePharma, Shanghai, China) to establish overexpression plasmids (OE-BMAL1, OE-USP10 or OE-HOXA5) with empty plasmids as the negative control (Vector). The specific siRNAs targeting BMAL1 were designed by GenePharma, named si-BMAL1, and the nontargeted siRNA was used as the contrast (si-NC). After confirming the transfection efficiency, the LPS exposure was performed in transfected cells.

### Animals model

Male C57BL/6 mice (8–10 weeks, 20–25 g, *n* = 5/each group) were purchased from Slaike Jingda Laboratory (Hunan, China). Animal Ethics Committee approved all animal procedures. The sepsis models were induced by cecal ligation and puncture (CLP). The caecum was exposed under aseptic conditions by a 2-cm midline laparotomy, and then ligated by a 4 − 0 silk suture, followed by puncturing using a 20-gauge needle for 2 times. All mice were injected 1 ml warm saline intraperitoneally and then placed in individual cages. In Sepsis + Ad-OE-NC or Sepsis + OE-BMAL1 group, mice were injected with adenovirus (Ad) containing OE-NC or OE-BMAL1 intravenously in the tail vein. All mice were anesthetized after CLP surgery at 12 h. The kidney tissues and blood were obtained for subsequent analyses.

### Histological examination

Paraffin-embedded kidney tissues were sectioned into slices with a thickness of 4-µm. For H&E staining, sections were deparaffinized using xylene and incubated with hematoxylin for a duration of 10 min, followed by dyeing with eosin for 5 min. For MASSON staining, sections were stained for 5 min with 1% Hematoxylin, washed with 95% ethanol for 2 times, and then dyed with acid ponceau solution for 1 min. Slides were visualized under a microscopy.

### Immunohistochemistry (IHC)

Paraffin-embedded kidney tissues were deparaffinized using xylene, hydrated in graded ethanol, followed by microwaving in EDTA for 15 min and then soaking in 3% hydrogen peroxide to quench for endogenous peroxidase. The slides were incubated with diluted primary antibody BMAL1 for at 4℃ for 12 h and secondary antibody for a half hour at 37℃. Following washing, the slides were visualized with diaminobenzidine and counterstained with hematoxylin. Slides were visualized under a microscopy.

### Renal function assessment

Blood samples were obtained from eyeballs and centrifuged at 4℃ (3000 rpm, 20 min. Then levels of blood urea nitrogen (BUN) and serum creatinine (Scr) were detected by a creatinine assay kit (Nanjing Jiancheng, Nanjing, China) and a colorimetric method.

### Western blotting

Total proteins were isolated by using the RIPA lysis buffer (Yeasen, Shanghai, China), then a BCA kit (Beyotime) was applied to determine protein concentration. Thereafter, 30 µg protein samples were loaded onto 10% SDS-PAGE gels for separation, followed by shifting onto nitrocellulose membranes. Membranes were then incubated with USP10 (ab70895), HOXA5 (ab140636), BMAL1 (ab230822), glutathione peroxidase 4 (GPX4) (ab41787), SLC7A11 (ab300667), and GAPDH (ab128915) (Abcam, Cambridge, UK) at 4℃ for 12 h, followed by reacting with Goat Anti-Rabbit or Mouse IgG H&L (Abcam) for 2 h at 37℃. Protein bands were analyzed using Image J software.

### Quantitative real time-PCR

The TRIzol (Pufei, Shanghai, China) was used to isolate total RNAs, then reverse transcription was performed, followed by qRT-PCR analysis with cDNA templates. Fold changes were tested by the 2^−ΔΔCt^ method and normalized to GAPDH expression. Table 1 exhibites the qRT-PCR primers.

**Table 1:** Primers for qRT-PCR

Name		Primers for qRT-PCR (5’−3’)
BMAL1	Forward	AAGCTGACCGCCTGAAAAGA
	Reverse	GTGCTCCCCAAATTCGACCT
GAPDH	Forward	TTTTGCGTCGCCAGCC
	Reverse	ATGGAATTTGCCATGGGTGGA

### MTT assay

Assigned HK-2 cells were seeded onto a 96-well plate at a quantity of 2.5 × 10^3^ cells/well. Each well was added with 20 µL MTT (5 mg/ml) (Beyotime). After 2 h of incubation, 150 µL DMSO was added. Finally, the absorbance at 450 nm was examined.

### 5-Ethynyl-2’-deoxyuridine (EdU) assay

Assigned HK-2 cells (5 × 10^4^ cells) in a 96-well plate was incubated with 100 µL of 50 µM EdU medium (RiboBio, Guangzhou, China) for 2 h. Then cells were washed with PBS for twice, fixed with 4% PFA (Beyotime) for a half hour and then permeabilized with 0.5% Triton X-100 for 10 min. Thereafter, cells were dyed with 100 µL 1×Apollo staining solution, followed by counterstaining with DAPI. At last, EdU-positive cells were examined using a fluorescence microscopy.

### Flow cytometry

Assigned HK-2 cells in each group were colleced and resuspended in 1×Annexin V binding buffer, after which they were incubated with 10µL Annexin V-FITC and 10µL propidium iodide (KeyGen, Nanjing, China) avoiding from light for 15 min. Apoptotic cells were finally examined using flow cytometry.

### ELISA analysis

Levels of IL-1β and IL-6 were analyzed by ELISA kits (R&D, Shenzhen, China). Cell supernatant was gathered from assigned HK-2 cells by centrifuging at 2000 g for 10 min, and then incubated with antibody cocktail for 1 h. Then samples and control buffer were added into the mixture and incubated with shaking for 1 h. 100 µL of Stop Solution was finally added to stop reaction. Lastly, the absorption was examined to calculate IL-1β and IL-6 levels.

### Reactive oxygen species (ROS) measurement

Assigned HK-2 cells were treated with 10 µM 2’,7’-dichlorofluorescein-diacetate (DCFH-DA, Sigma-Aldrich) under darkness at 37℃for 30 min. Thereafter, fluorescent densities was detected at 488/525 nm.

### Measurement of iron ion

An iron assay kit (Abcam) was used for this measurement. Assigned HK-2 cells were lysed and cell supernatant was collected by centrifugation. Thereafter, cell supernatant was incubated with the assay buffer for 30 min, and then reacted with iron probe for 1 h. Lastly, intracellular Fe2 + concentration was calculated by reading the absorbance at 593 nm.

### Measurement of malondialdehyde (MDA)

A MDA assay kit (Nanjingjiancheng) was applied for MDA level examination in the supernatant of assigned HK-2 cells following the recommended instruction. The absorbance was examined at 532 nm to assess MDA production.

### Co-immunoprecipitation (Co-IP) assay

The protein-protein interaction was detected by Co-IP assay. HK-2 cells were lysed by 500 µl co-IP buffer mixture, and then centrifuged at 12,000 g for 30 min. Cell supernatant was collected and incubated with beads and corresponding antibodies overnight. After washing, the immunoprecipitates were subjected to western blotting.

### Ubiquitination assay

 293 T cells were overexpressed USP10 using OE-USP10 plasmids. 48 h later, cells were collected and lysed in RIPA buffer, and BMAL1 was first immunoprecipitated using protein A/G magnetic beads (Thermo Fisher Scientific, Inc., Waltham, MA, USA) with anti-BMAL1. Then the ubiquitination of BMAL1 was immunoblotted by western blotting using ubiquitin antibody (ab134953, Abcam).

### Chromatin Immunoprecipitation (ChIP) assay

The binding of HOXA5 to BMAL1 promoter was analyzed using an Agarose ChIP kit (Thermo Fisher). Briefly, the crosslinking of HK-2 cells was processed for 10 min by 1% formaldehyde, which was followed terminated using 0.125 M Glycine (Thermo Fisher). Then cells were lysed and chromatins were sonicated to yield DNA fragments (200–500 bp), followed by incubating with anti-HOXA5 (abcam) for 12 h with the anti-IgG (Millipore) as the input control. After being incubated for 6 h with the Protein A/G-agarose beads, beads were eluted and crosslinking was reversed via incubation at 65℃ for 4 h. The purified immunoprecipitated DNA was amplified by qRT-PCR.

### Luciferase reporter assay

The wild-type of HOXA5-binding sites to BMAL1 promoter was separately subcloned into pGL3 luciferase reporter vector (Promega, Madison, USA) to generate WT-BMAL1 luciferase reporters. Then seed region of HOXA5 binding sites was mutated and MUT-BMAL1 vector was generated. Then above vectors were co-transfected into HK-2 cells with OE-HOXA5 or vector. 48 h later, a dual Luciferase Assay kit (Promega) was for assaying the luciferase activity.

### Statistical analysis

Data are given as means ± standard deviation (SD). Three independent biological replicates were performed in each experiment. The normal distribution and similarity of variance were analyzed by Shapiro-Wilk Normality Test and Bartlett’s test. For multiple group comparisons, analysis of variance followed by Tukey’s post-test was performed. Statistical analyses in two groups were carried out using Student’s t test. *P* < 0.05 was defined as statistically significant difference.

## Results

### BMAL1 overexpression reverses LPS-induced apoptosis, inflammation and ferroptosis in HK-2 cells

**Fig. 1:**
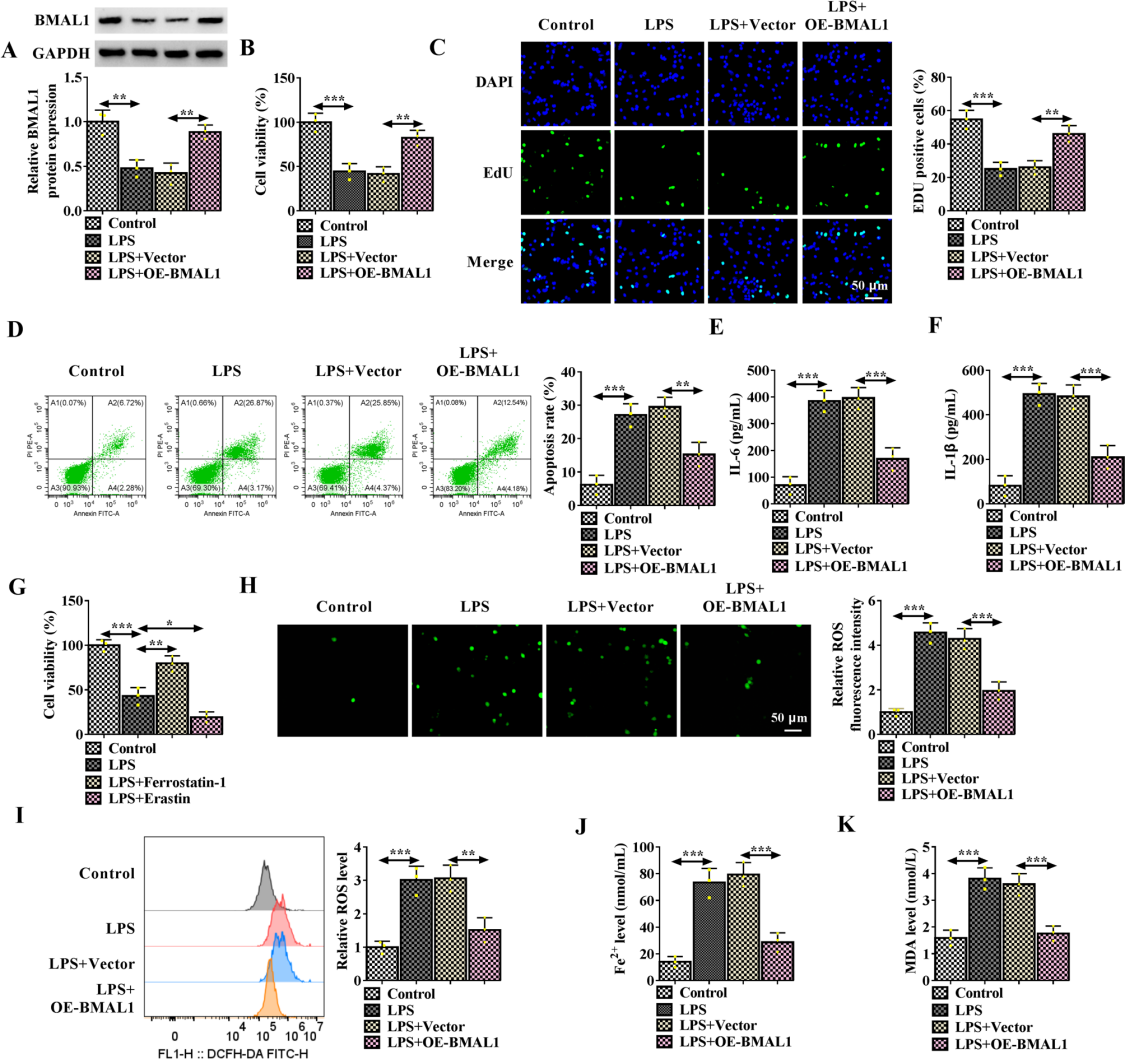
BMAL1 overexpression reverses LPS-induced apoptosis, inflammation and ferroptosis in HK-2 cells. **A**-**K** HK-2 cells were transfected with OE-BMAL1 or Vector, followed by LPS treatment. **A** Western blotting analysis for BMAL1 expression in HK-2 cells after transfection. **B**, **C** Cell proliferation analysis using MTT and EdU assays. **D** Flow cytometry for cell apoptosis. **E**, **F** ELISA analysis for the levels of IL-6 and IL-1β. **G** MTT assay for the viability of LPS-treated HK-2 cells after the treatment of Ferrostain-1 or Erasin. **H**, **I** Detection of ROS generation by DCFH-DA staining. **J**, **K** Measurement of the Fe2 + and MDA levels in cells by kits. *P < 0.05, **P < 0.01, ***P < 0.001

To investigate the role of BMAL1 in sepsis-induced AKI, in vitro experiments using LPS-induced HK-2 cell models were conducted. BMAL1 was overexpressed in HK-2 cells with OE-BMAL1 vector, then cells were treated with LPS. Western blotting analysis showed that BMAL1 expression was decreased in LPS-exposed HK-2 cells, which were then increased by OE-BMAL1 (Fig. 1A). Functionally, it was found that BMAL1 overexpression reversed LPS-induced suppression of HK-2 cell proliferation (Fig. 1B, C) and promotion of cell apoptosis (Fig. 1D). In addition, proinflammatory cytokines IL-6 and IL-1β were found to be increased under LPS treatment in HK-2 cells, while their levels were decreased by BMAL1 overexpression (Fig. 1E, F). In addition, it was observed that Ferrostain-1, an inhibitor of ferroptosis, could restrain LPS-induced proliferation inhibition in HK-2 cells, while Erasin, a ferroptosis activatior, was found to enhance LPS-induced inhibition of HK-2 cell proliferation (Fig. 1G), indicating that LPS might induce ferroptosis in HK-2 cells. Then we investigated whether BMAL1 was involved in LPS-induced ferroptosis. It was found that BMAL1 overexpression reduced LPS-induced increase in ROS levels in HK-2 cells (Fig. 1H, I). In addition, Fe2 + levels were up-regulated by LPS treatment in HK-2 cells, which were reduced by BMAL1 overexpression (Fig. 1J). Moreover, results showed that LPS treatment led to the increase of MDA generation in HK-2 cells, while this increase was reduced by BMAL1 overexpression (Fig. 1K). Low activity or inactivation of GPX4 is identified as the specific marker of ferroptosis, besides that, SLC7A11 is important in preventing ferroptosis [14]. Herein, it was also observed that BMAL1 overexpression rescued LPS-induced decreases of the protein expression of GPX4 and SLC7A11 (Fig. S1A, B). These results suggested that BMAL1 overexpression reversed LPS-induced ferroptosis in HK-2 cells.

### USP10 stabilizes BMAL1 expression in HK-2 cells by deubiquitination

**Fig. 2:**
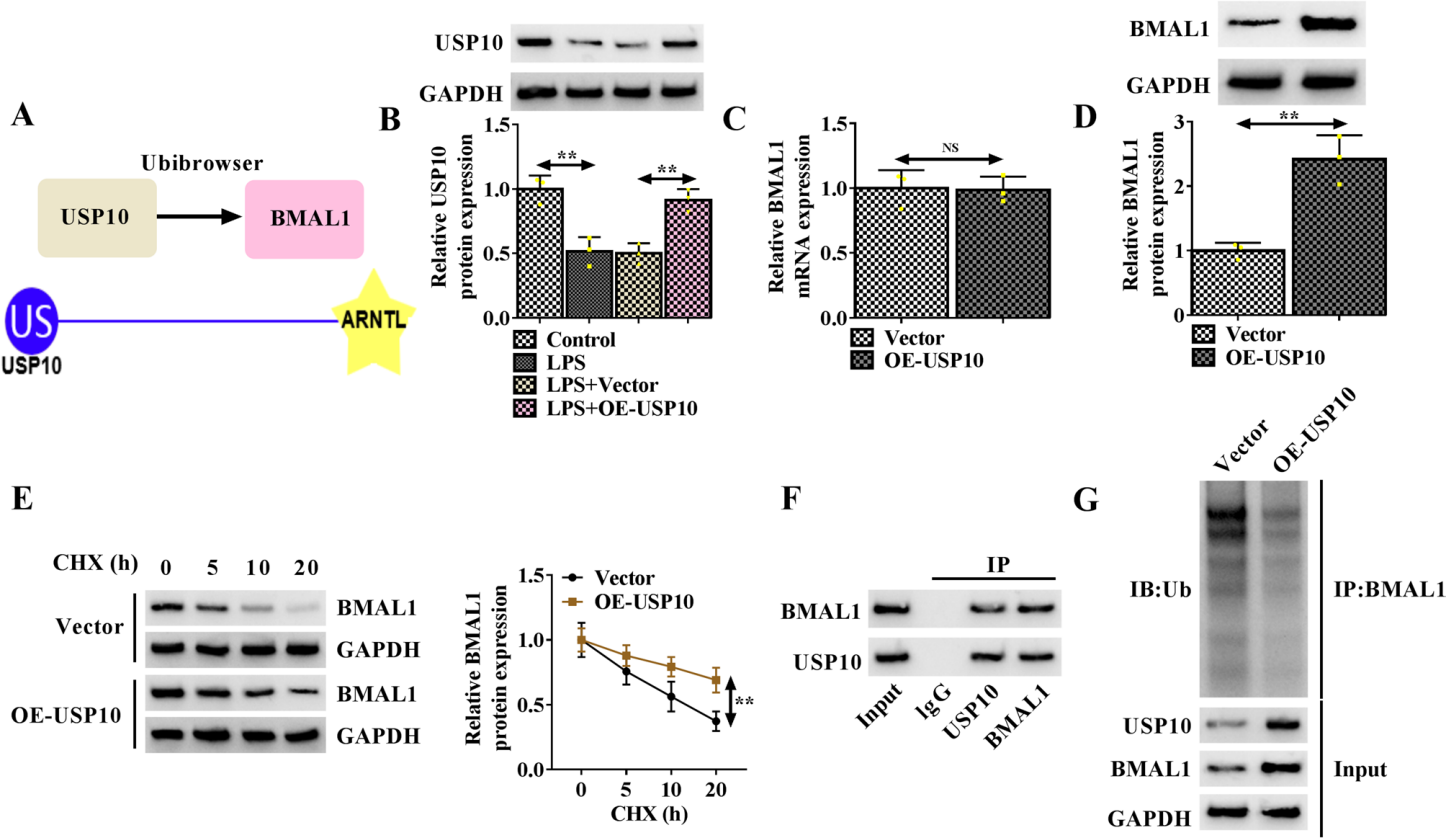
USP10 stabilizes BMAL1 expression in HK-2 cells by deubiquitination. **A** Ubibrowser website shows that USP10 may modulate BMAL1 expression by deubiquitination. **B** Detection of USP10 protein expression in LPS-treated HK-2 cells after transfecting with OE-USP10 or Vector. **C**, **D** Levels of USP10 mRNA and protein were detected in HK-2 cells after OE-USP10 or Vector transfection. **E** BMAL1 protein levels in HK-2 cells transfected with OE-USP10 or Vector were evaluated by western blotting in the presence of CHX for indicated time point. **F** Co-IP assay for the protein binding between USP10 and BMAL1. **G** Analysis of USP10, BMAL1 protein and ubiquitination levels by Western blot in 293 T transfected with OE-USP10 or Vector. **P < 0.01

Subsequently, we explore the upstream mechanism of BMAL1 in regulating LPS-induced injury. Ubibrowser website shows that USP10 may modulate BMAL1 by deubiquitination (Fig. 2A). USP10 expression was decreased by LPS in HK-2 cells, while its expression was rescued by OE-USP10 vector (Fig. 2B). Then we found that USP10 overexpression did not affect BMAL1 mRNA expression, but caused an increase of BMAL1 protein expression (Fig. 2C, D). Furthermore, it was observed that USP10 overexpression significantly prolonged the half-life of BMAL1 in the presence of cycloheximide (CHX), a protein synthesis inhibitor, in HK-2 cells (Fig. 2E). The protein binding between BMAL1 and USP10 was verified by Co-IP assay (Fig. 2F). Besides that, USP10 overexpression notably reduced the ubiquitination of BMAL1 in 293 T cells (Fig. 2G).

### USP10 overexpression reverses LPS-induced apoptosis, inflammation and ferroptosis in HK-2 cells by regulating BMAL1

**Fig. 3:**
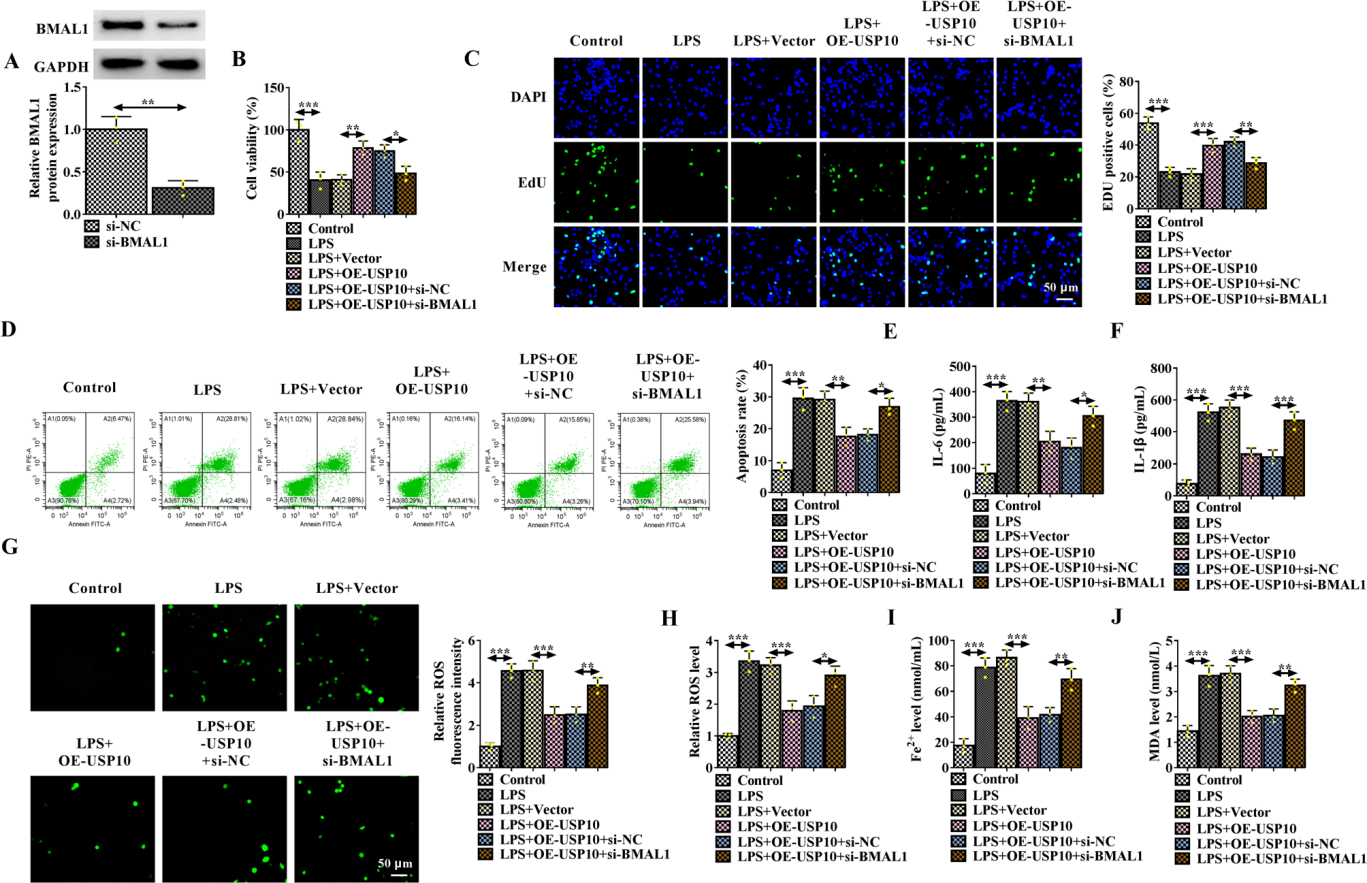
USP10 overexpression reverses LPS-induced apoptosis, inflammation and ferroptosis in HK-2 cells by regulating BMAL1. **A** The interference efficiency of si-BMAL1 or si-NC was detected by western blotting. **B**-**J** HK-2 cells were transfected with Vector, OE-USP10, OE-USP10 + si-NC, or OE-USP10 + si-BMAL1, followed by LPS treatment. **B**, **C** Cell proliferation analysis using MTT and EdU assays. **D** Flow cytometry for cell apoptosis. **E**, **F** ELISA analysis for the levels of IL-6 and IL-1β. **G**, **H** Detection of ROS generation by DCFH-DA staining. **I**, **J** Measurement of the Fe2 + and MDA levels in cells by kits. *P < 0.05, **P < 0.01, ***P < 0.001

Subsequently, the function of USP10 in sepsis-induced AKI was explored. The si-BMAL1 was first designed, and western blotting showed that levels of BMAL1 were markedly decreased by si-BMAL1 transfection compared with the control si-NC in HK-2 cells (Fig. 3A). Functionally, USP10 overexpression reversed LPS-induced proliferation suppression (Fig. 3B, C) and apoptosis promotion (Fig. 3D) in HK-2 cells, while these effects were reversed by BMAL1 silencing (Fig. 3B-D). In addition, levels of IL-6 and IL-1β were decreased by USP10 overexpression in LPS-induced HK-2 cells, but were increased after BMAL1 silencing (Fig. 3E, F). Moreover, USP10 overexpression reduced LPS-induced generation of ROS, Fe2+, and MDA, which were rescued by BMAL1 silencing (Fig. 3G-J). Besides that, USP10 overexpression rescued LPS-induced decreases of GPX4 and SLC7A11 protein expression in HK-2 cells, while this increase of GPX4 and SLC7A11 protein expression mediated by USP10 was reduced by BMAL1 silencing (Fig. S1A, B).

### HOXA5 induces the transcription of BMAL1

**Fig. 4:**
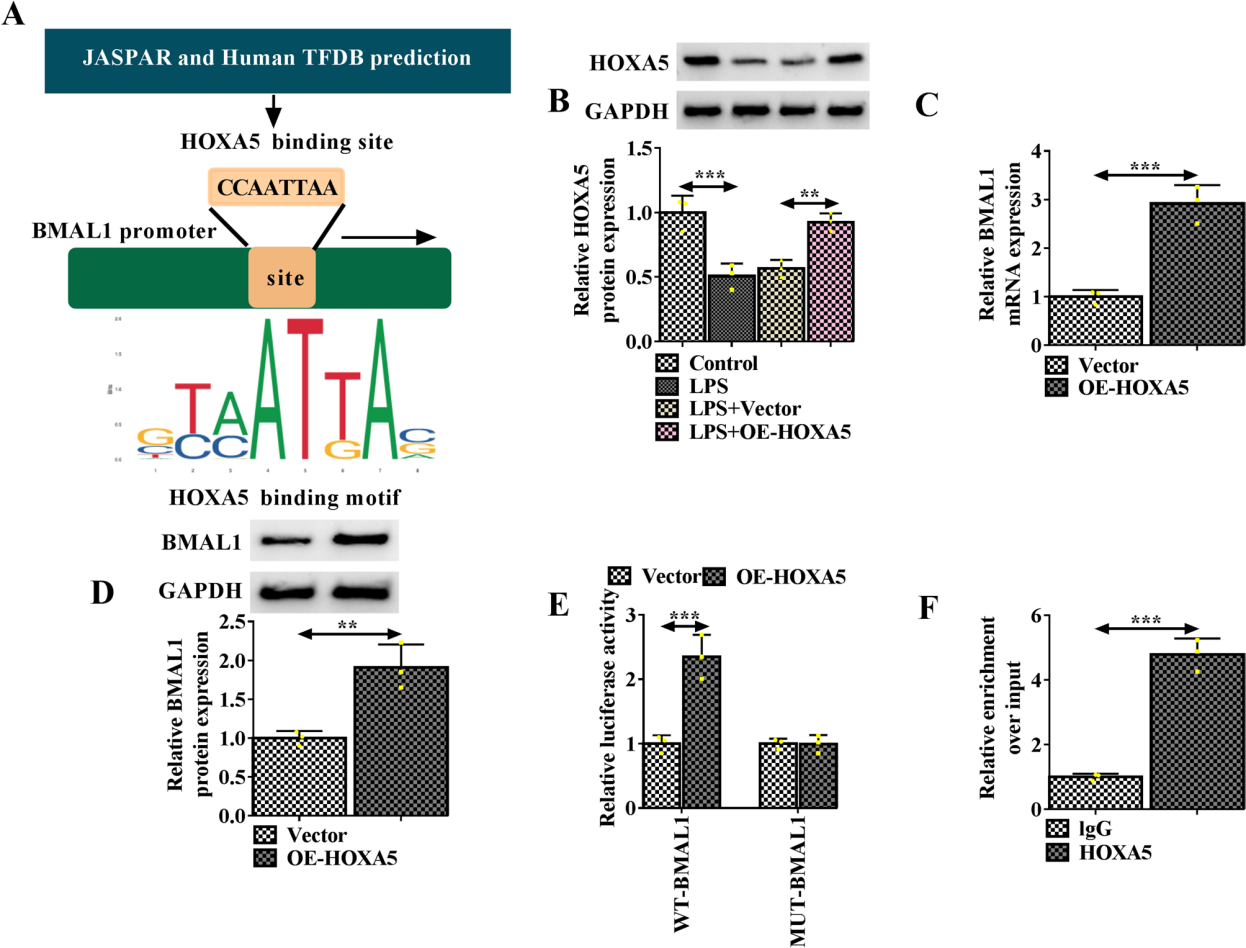
HOXA5 induces the transcription of BMAL1. **A** HOXA5 is predicted to have binding sites on the promoter of BMAL1. **B** Western blotting analysis for HOXA5 in LPS-induced HK-2 cells after OE-HOXA5 or Vector transfection. **C**, **D** qRT-PCR and Western blotting analysis for BMAL1 expression in HK-2 cells after HOXA5 overexpression. **E**, **F** Luciferase reporter assay and ChIP assay were used to verify the binding between HOXA5 on the promoter region of BMAL1. **P < 0.01, ***P < 0.001

Next, it is found that HOXA5 has binding sites on the promoter of BMAL1 (Fig. 4A). HOXA5 expression was decreased in LPS-induced HK-2 cells, and was rescued by OE-HOXA5 vector (Fig. 4B). Then it was found that HOXA5 overexpression caused the increase of BMAL1 expression both at mRNA and protein levels in HK-2 cells (Fig. 4C, D). Next, results of luciferase reporter assay showed that HOXA5 overexpression increased the luciferase activity of WT-BMAL1 reporter but not the MUT-BMAL1 reporter (Fig. 4E). ChIP assay also verified that HOXA5 bound to the promoter region of BMAL1 (Fig. 4F).

### HOXA5 overexpression reverses LPS-induced apoptosis, inflammation and ferroptosis in HK-2 cells by regulating BMAL1

**Fig. 5:**
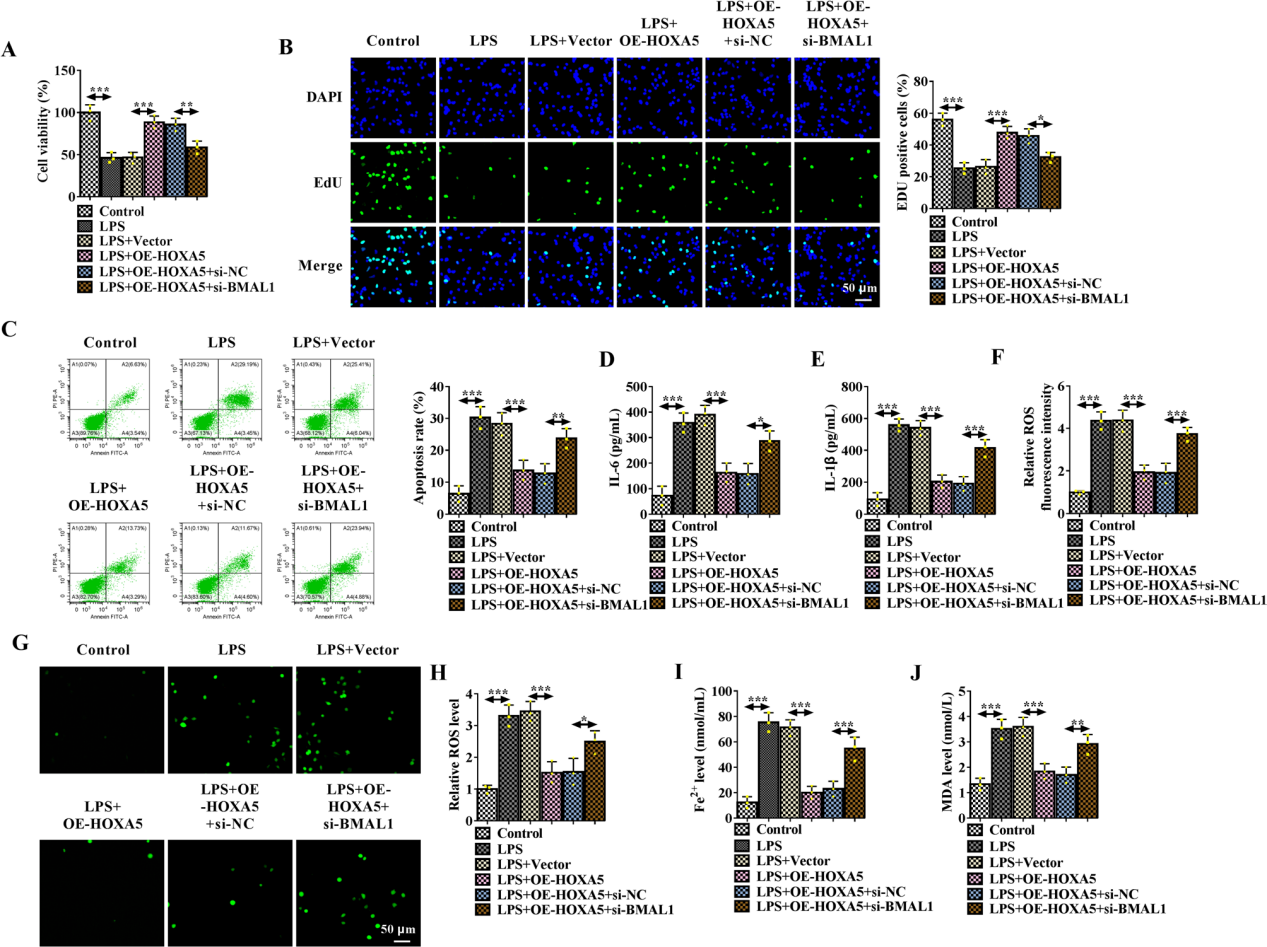
HOXA5 overexpression reverses LPS-induced apoptosis, inflammation and ferroptosis in HK-2 cells by regulating BMAL1. **A**-**J** HK-2 cells were transfected with OE-HOXA5 or OE-HOXA5 and si-BMAL1, followed by LPS treatment. **A**, **B** Cell proliferation analysis using MTT and EdU assays. **C** Flow cytometry for cell apoptosis. **D**, **E** ELISA analysis for the levels of IL-6 and IL-1β. (F-H) Detection of ROS generation by DCFH-DA staining. **I**, **J** Measurement of the Fe2 + and MDA levels in cells by kits. *P < 0.05, **P < 0.01, ***P < 0.001

Thereafter, the function of HOXA5 in sepsis-induced AKI was investigated. HK-2 cells were transfected with OE-HOXA5 or OE-HOXA5 and si-BMAL1, followed by LPS treatment. Functionally, HOXA5 overexpression abated LPS-induced proliferation suppression (Fig. 5A, B) and apoptosis promotion (Fig. 5C) in HK-2 cells, while these effects were rescued by BMAL1 decrease (Fig. 5A-C). In addition, the increase of IL-6 and IL-1β levels caused by LPS was reduced by HOXA5 overexpression, but increased after BMAL1 silencing (Fig. 5D, E). Besides that, HOXA5 overexpression decreased LPS-induced generation of ROS, Fe2+, and MDA, which were rescued by BMAL1 silencing (Fig. 5F-J). Moreover, the decreases of GPX4 and SLC7A11 protein expression caused by LPS were rescued after HOXA5 overexpression, while these increases were reduced by BMAL1 silencing (Fig. S1C, D).

### BMAL1 overexpression attenuates sepsis-induced AKI in mouse models

**Fig. 6:**
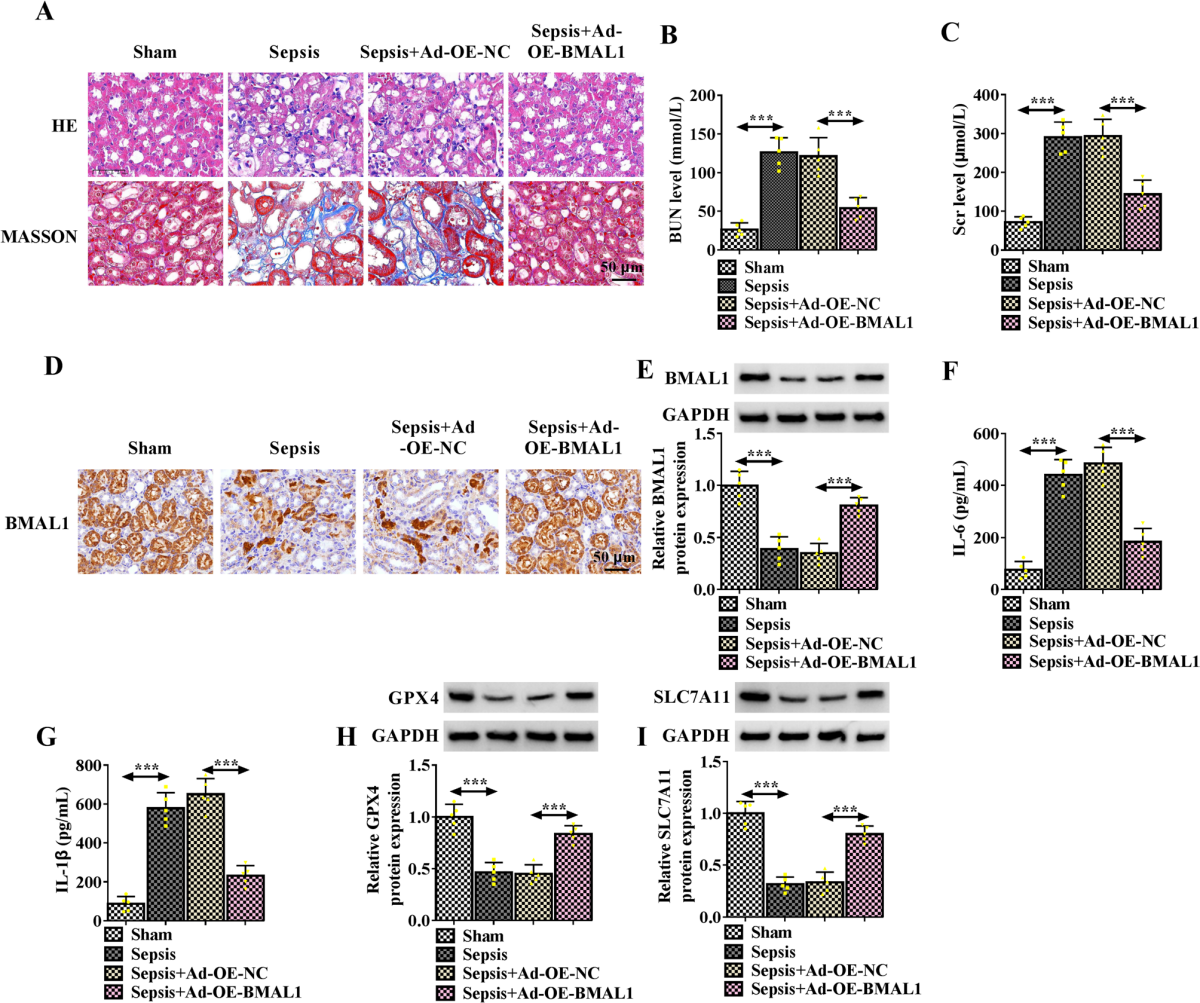
BMAL1 overexpression attenuates sepsis-induced AKI in mouse models. **A** H&E staining and Masson staining were applied to observe the histological changes of kidney tissues in septic mouse models. (**B**, **C**) Blood samples were collected for the quantification of BUN and Scr. **D**, **E** IHC analysis and western blotting for BMAL1 expression in kidney tissues of septic mouse models. **F**, **G** ELISA analysis for IL-1β and IL-6 levels in the serum of each group. **H**, **I** Western blotting for the protein levels of GPX4 and SLC7A11 in kidney tissues of septic mouse models. ***P < 0.001

To investigate the role of BMAL1 in sepsis-induced AKI, mouse sepsis models were established. In the results from H&E and Masson staining, compared with the sham group, there was the loss of brush border, vacuolar degeneration, and cast formation of kidney tissues in the model group, while these histological changes could be ameliorated by BMAL1 overexpression (Fig. 6A). In addition, both the BUN and Scr expression levels were markedly increased in the septic mouse models, while their expression was reduced in models with high BMAL1 (Fig. 6B, C). Thereafter, it was observed that BMAL1 expression was decreased in kidney tissues of septic mouse models, but its level was reduced in kidney tissues of BMAL1-overexpressed septic mouse models (Fig. 6D, E). Moreover, levels of IL-6 and IL-1β were higher in septic mouse models, while were reduced in BMAL1-overexpressed septic mouse models (Fig. 6F, G). Herein, it was also observed that protein levels of GPX4 and SLC7A11 was decreased in kidney tissues of septic mouse models, but was rescued by BMAL1 overexpression (Fig. 6H, I).

## Discussion

Herein, it was confirmed that BMAL1 elevation neutralized LPS-triggered apoptosis, inflammation and ferroptosis in HK-2 cells in vitro, importantly, BMAL1 overexpression could recover renal function and reduce the histological damages of kidney tissues in septic mouse models. Then the mechanisms by which BMAL1 overexpression need to be better understood. We further demonstrated that USP10 bound to BMAL1 and positively modulated BMAL1 protein expression by reducing BMAL1 ubiquitination. Moreover, HOXA5 acted as the transcription factor to induce BMAL1 transcription, thereby increasing its expression.

Ubiquitination is a significant post-translational modification that covalently attaching ubiquitin (Ub) to a target protein, thereby modulating the functional activity and stability of proteins [15]. Both normal and abnormal conditions, around 90% of intracellular proteins are degraded via the ubiquitin-proteasome protein-degradation system [16, 17]. Ubiquitination is able to be reversed by deubiquitinating enzymes (DUBs) [18, 19]. As the member of USPs, the largest group of DUBs, USP10 has been reported to have enormous significance in various biological processes [20, 21]. Moreover, USP10 also affected immune-associated signaling pathways through reducing the ubiquitin of immune-related genes [22]. In sepsis, Gao et al. showed that USP10 attenuated sepsis-induced AKI in mouse models and suppressed LPS-induced oxidative stress and apoptosis in renal tubular epithelial cells through activating the NRF2/ARE signaling via SIRT6 [23]. USP10 deubiquitinated FOXQ1 to stabilize its protein expression, which then alleviated sepsis AKI-evoked apoptosis and inflammation [24]. Herein, it was confirmed that USP10 stabilized BMAL1 protein expression in HK-2 cells by deubiquitination, moreover, USP10 elevation neutralized LPS-triggered apoptosis, inflammation and ferroptosis in HK-2 cells, which were rescued by BMAL1 deficiency. In addition, it was also confirmed that HOXA5 transcription factor induced BMAL1 transcription. Wang et al. showed that HOXA5 upregulation mediated by SIRT5-induced desuccinylation attenuated LPS-evoked ferroptosis suggesting the potential involvement of HOXA5 in septic lung injury [25]. It was confirmed that interrupting neutrophil extracellular traps formation could enhance the augment efficacy of anti-Fn14 therapy in sepsis-associated AKI by dictating the stability of tubular HOXA5 [26]. Herein, it was also demonstrated that HOXA5 overexpression abated LPS-evoked apoptosis, inflammation and ferroptosis in HK-2 cells, while the protective effects were reversed by BMAL1 deficiency. However, there are still some shortcomings. The upstream signaling responsible for HOXA5 decrease upon LPS stimulation and the relationship between HOXA5 and USP10 in regulating BMAL1 under LPS treatment remain unclear. These still need us to conduct further study.

In conclusion, BMAL1 overexpression mediated by USP10-induced deubiquitination or HOXA5-induced transcription could attenuate sepsis-induced acute kidney injury. BMAL1 is an immune system regulator that governs phagocytic responses after infection [27]. Thus, antibiotics combined with BMAL1 agonist may be potential treatment approach for sepsis AKI with a special focus on the immune system. In addition, BMAL1 functions as a ferroptosis inhibitor [28]. Ferroptosis can trigger the first wave of death, inducing an inflammatory response that in turn contribute to the deterioration of renal function [29]. Targeting BMAL1 shows great therapeutic potential for sepsis AKI by modulating ferroptosis. Therefore, this study suggests a novel insight for the prevention of sepsis-induced AKI.

## Supplementary Information

Supplementary Material 1

Supplementary Material 2

## Data Availability

The datasets are available from the corresponding author on reasonable request.
