# Supplementary material for: BMAL1 alleviates sepsis-induced acute kidney injury by inhibiting apoptosis, ferroptosis and inflammation
Source: Hereditas. 2025 Oct 14;162:208. doi: 10.1186/s41065-025-00583-5 (PMC12522825; doi:10.1186/s41065-025-00583-5)
Supplement: Supplementary file 1 — Supplementary Material 1 [file 41065_2025_583_MOESM1_ESM.pdf]

The original western blots of Fig1

A

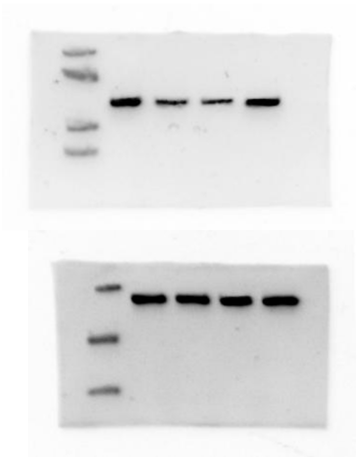

BMAL1

GAPDH

The original western blots of Fig2

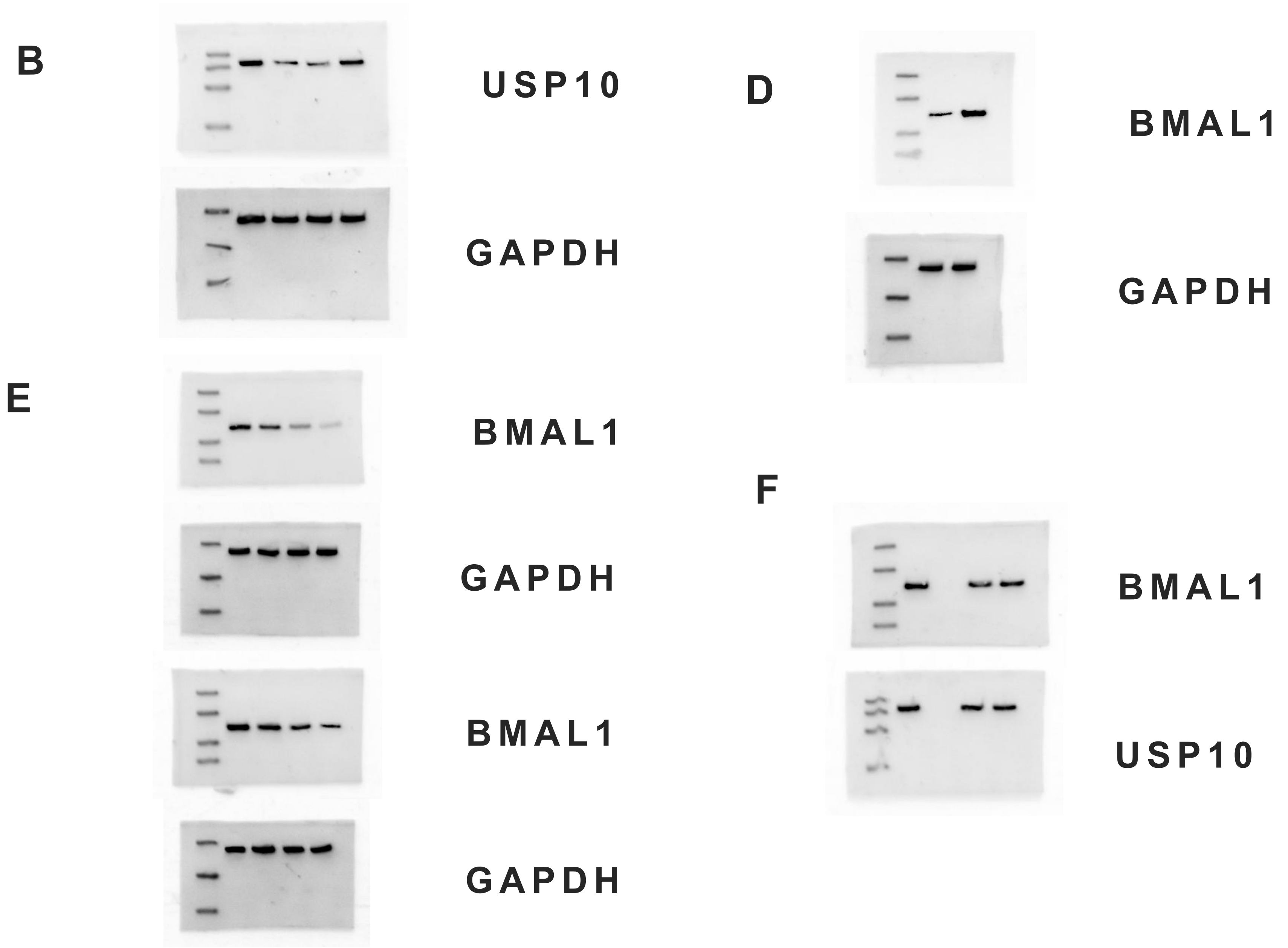

The original western blots of Fig2

G

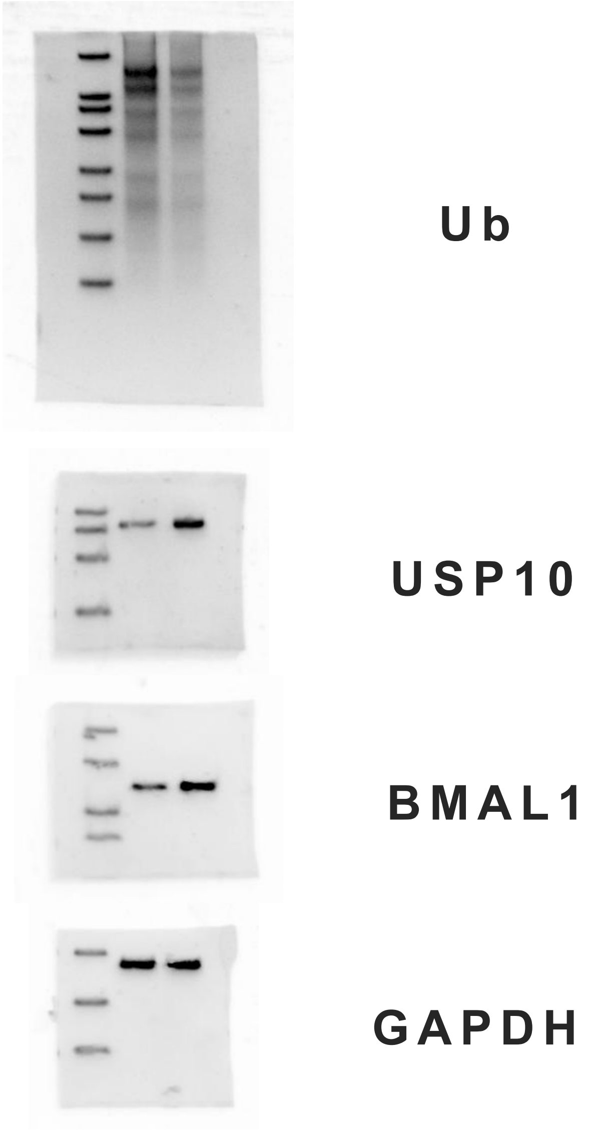

The original western blots of Fig3

A

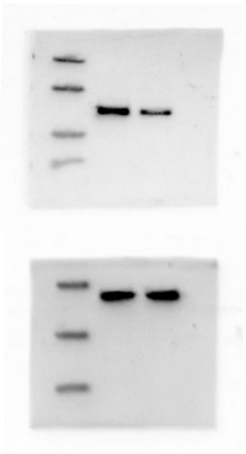

BMAL1

GAPDH

The original western blots of Fig4

B

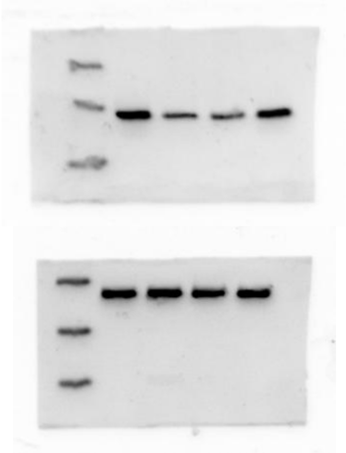

HOXA5

GAPDH

D

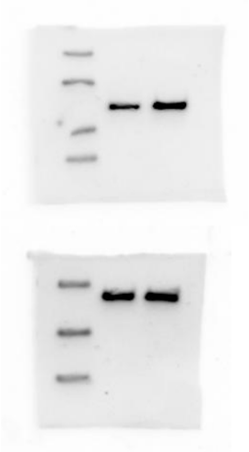

BMAL1

GAPDH

The original western blots of Fig6

E

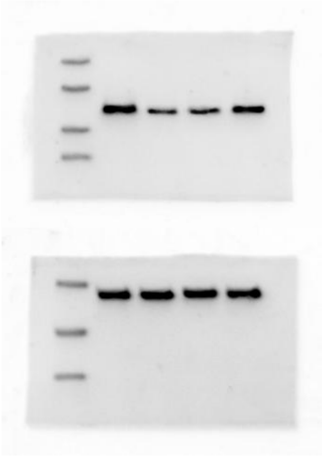

BMAL1

GAPDH

H

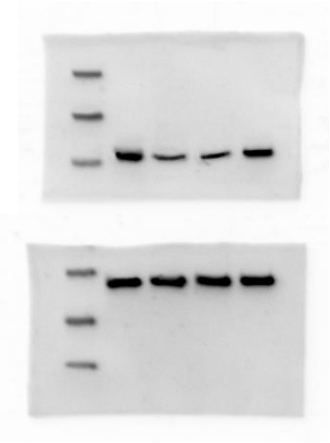

GPX4

GAPDH

I

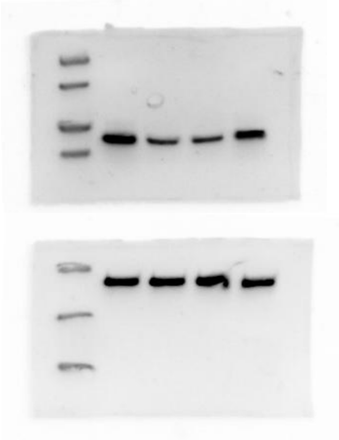

SLC7A11

GAPDH
