# Supplementary figures and images for: BMAL1 alleviates sepsis-induced acute kidney injury by inhibiting apoptosis, ferroptosis and inflammation
Source: Hereditas. 2025 Oct 14;162:208. doi: 10.1186/s41065-025-00583-5 (PMC12522825; doi:10.1186/s41065-025-00583-5)

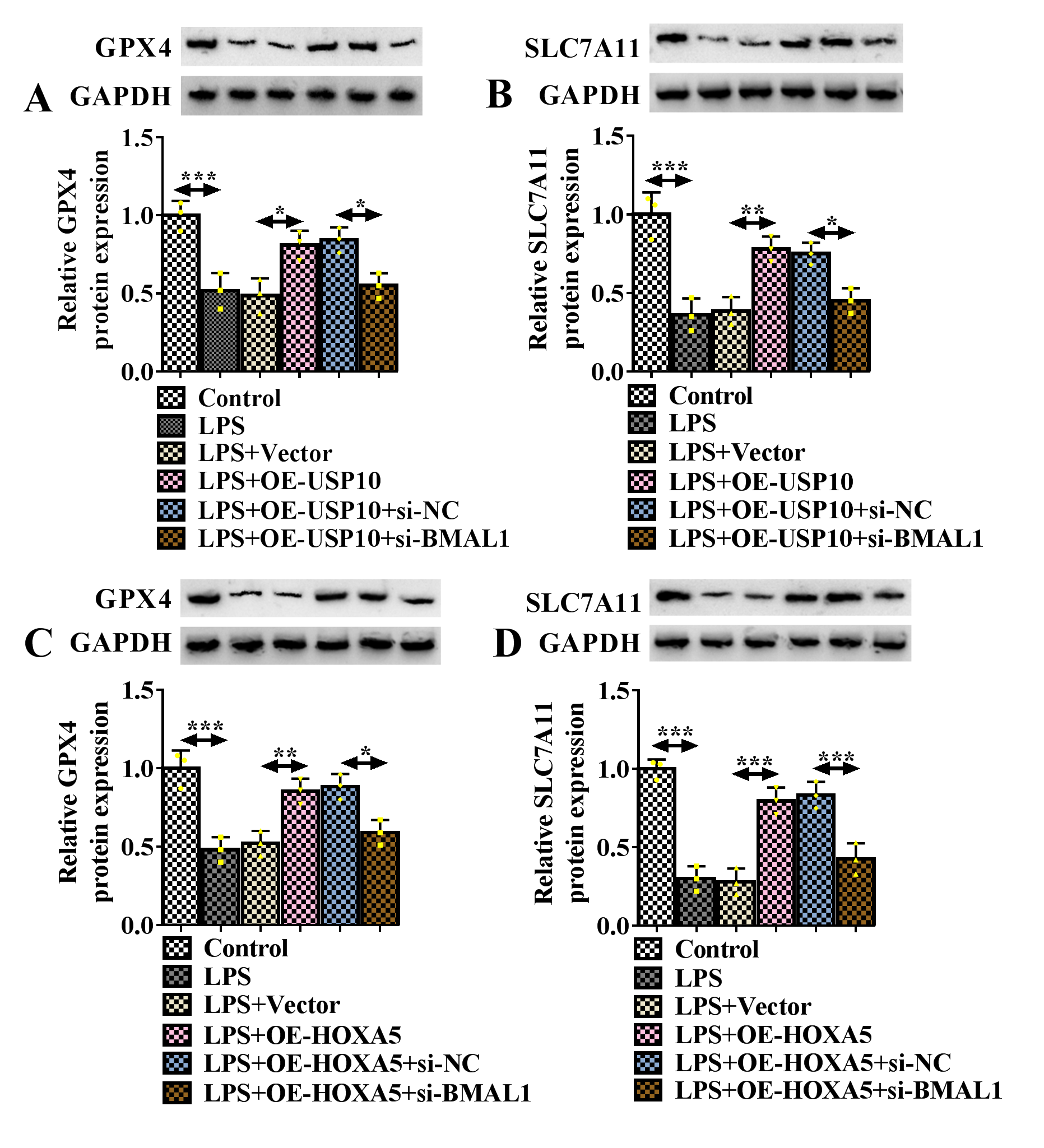

Supplement: Supplementary file 2 — Supplementary Material 2 [file 41065_2025_583_MOESM2_ESM.tif]
